# Dietary Supplement Use in Competitive Spanish Football Players and Differences According to Sex

**DOI:** 10.3390/nu17040602

**Published:** 2025-02-07

**Authors:** Carlos Sousa-Rufino, Helios Pareja-Galeano, María Martínez-Ferrán

**Affiliations:** 1 Faculty of Health Sciences, Universidad Isabel I, 09003 Burgos, Spain; carlossousarufino@gmail.com; 2 Department of Physical Education, Sport and Human Movement, Universidad Autónoma de Madrid, 28049 Madrid, Spain; 3 Faculty of Health Sciences, Universidad de Burgos, 09001 Burgos, Spain; mmferran@ubu.es

**Keywords:** dietary supplements, football, soccer, nutrition, ergogenic aids, performance

## Abstract

**Objectives**: This study aimed to evaluate and compare the consumption of dietary supplements (DS) in semi-professional male and professional female Spanish football players. **Methods**: The study involved 129 football players (92 men and 37 women) who completed a validated, self-administered questionnaire on the use of DS in athletes. **Results**: The results indicated that 96.12% of the sample reported taking DS, with the main reason for consumption being performance enhancement. The most commonly consumed DS were creatine monohydrate (66.77%), caffeine (56.59%), whey protein (48.06%), isotonic drinks (37.21%), vitamin D (27.91%), energy bars (27.13%), magnesium (20.93%), and vitamin C (20.16%). Additionally, significant differences were found between sexes regarding vitamin D consumption (*p* < 0.05; OR = 3.27 [0.36–2.00]). According to the Australian Institute of Sport DS classification, group A was the most consumed, followed by group B. Significant sex differences were found in the consumption of sports foods (*p* = 0.034; OR = 3.25 [0.05–2.31]) and medical supplements (*p* < 0.001; OR = 3.75 [0.52–2.12]). Sex differences were also found in place of purchase, source of recommendation, as well as the situation and timing of consumption (*p* < 0.05). **Conclusions**: The use of DS among Spanish football players was prevalent at 96.12%, with creatine monohydrate, caffeine, and whey protein being the most commonly consumed. Differences in consumption patterns were noted between sexes.

## 1. Introduction

Football is the most popular and widely played sport in the world and is constantly evolving. In both training and competition, the physical and technical demands of today’s football have evolved considerably, with more tactically and physically demanding training sessions, a fixture schedule with a greater volume of matches, an increased variability in schedules due to the influence of television broadcasts, an increased number of trips, and the great cultural diversity of the players who make up the squads [1,2].

In terms of metabolic demands, football, due to its intermittent nature, requires a large number of actions involving submaximal and maximal efforts with a high mechanical and metabolic load. Football players must therefore have a high capacity for aerobic and anaerobic energy production that allows them to perform repeated high-intensity actions with recovery periods in between, as well as the total distance covered. This increasingly requires players who are fast, skilled, and have a body composition with a high muscle-to-fat ratio [3].

The development and increased demands of modern football lead to the development of muscular fatigue and a consequent reduction in athletic performance. This fatigue can be temporary during periods of intense exertion and is particularly pronounced in the closing stages of matches when the outcome of a match can be decided [3,4].

In addition, in elite football, the total number of matches played in a season can be as high as 60 between competitions. This is a major nutritional challenge, as an appropriate nutritional strategy must be implemented to provide sufficient nutrient intake to support the adaptations to training and competition, which can reduce the risk of injury and recovery time. Therefore, nutritional planning for football players should be individualised and adapted to training periods and different phases of the season [5].

Therefore, dietary supplements (DS) can play an important role in the nutritional strategy of football players, as their use promotes the supply of specific nutrients and helps to meet energy and macronutrient or micronutrient requirements that are difficult to achieve through food intake alone. In addition to being effective in addressing nutritional deficiencies, they help to maintain good health, prevent injuries, and directly improve athletic performance [6,7]. There are different classifications of DS, one of the best known of which is the ABCD system of the Australian Institute of Sport (AIS), which divides DS according to their effectiveness, safety, and legality, in which a risk–benefit analysis is carried out for each item. Thus, group A (subdivided into medical supplements, ergogenic aids, and sports foods) is the group with a high level of scientific evidence for improving health or performance in athletes; group B includes DS with possible positive effects that still need more evidence; group C includes DS with conclusive evidence against their use; and group D includes banned substances [8].

Additionally, it is important to raise awareness of the appropriate use of DS among football players and their support team (coach, physical trainer, nutritionist, doctor…) to provide them with information to help them make informed decisions. It is also necessary to understand the pros and cons of the use of sports supplements/foods, taking into account whether they are safe, effective, and permitted for use in sport [1,6].

The use of DS is widespread in all sports, although the prevalence varies from sport to sport [9,10,11]. In the case of football, the literature on the use of DS in football players is scarce, with very few studies providing data [2,12,13,14]. Moreover, considering the research gap in nutritional strategies for female football players and the importance of understanding their health and performance needs, we aim to address sex-based differences in dietary supplement (DS) use [15]. Therefore, the aim of this study was to evaluate the prevalence and consumption patterns of DS among football players and to analyse possible differences according to sex.

## 2. Materials and Methods

### 2.1. Participants and Experimental Design

A cross-sectional observational study was conducted on a sample of 129 football players (92 males and 37 females) from different Spanish football teams. Two participants answered the questionnaire incorrectly, so their results were excluded. The leagues included in the study were men’s semi-professional leagues (“Segunda Federación” of Spanish Football Federation, RFEF: Groups 1, 3, and 4) and women’s professional leagues (“Primera División Nacional de Fútbol Femenino”).

The characteristics of the sample are detailed in Table 1. Overall, 96.12% of the participants had been federated for more than ten years (men: 97.83%; women: 91.89%).

Non-probabilistic convenience sampling was used to select participants. The technical staff of the different teams (coaches and nutritionists) and the players themselves were contacted via e-mail and social networks to inform them of the characteristics of the study, its objectives, and to ask for their cooperation in participating in the study. Where recruitment was carried out by the clubs’ own coaching staff, they informed the players of the aims of the study. Players were also contacted directly to recruit other participants. Participation was voluntary and, once accepted, the players were sent an email with a link to the DS consumption questionnaire, which was completed electronically and anonymously.

Participants completed the study on a voluntary basis and gave their informed consent. They completed the questionnaire between March 2023 and June 2023 in a web-based format (Google Forms, Google, Mountain View, CA, USA). The questionnaires were completed anonymously, without the possibility of identifying participants.

The total number of male footballers in the Segunda Federación was estimated using competition regulations, which allow up to 22 players per team across 90 teams, resulting in 1980 players. For female footballers in the Primera División Nacional de Fútbol Femenino, regulations allow up to 25 players per team across 16 teams, resulting in 400 players. Thus, the combined population is estimated at 2380 players. The sample size recruited of 129 meets the requirements for representativeness with a 95% confidence level and a 9% margin of error, where 113 subjects are needed.

The protocol for this study adhered to the Declaration of Helsinki for research involving human subjects and was approved by the institutional ethics committee (approved on 27 February 2023 by the Ethics Committee of the Universidad Isabel I, in Burgos, Spain).

### 2.2. Instruments

A specific and validated DS intake questionnaire was used for data collection, which has been used several times in different sports settings [10,11,16,17,18,19,20,21] and was developed and validated by a group of experienced sports scientists [22]. In addition, its methodological quality was evaluated in a systematic review and meta-analysis [9]. Quality was measured using an 8-point scale assessing sampling methods, sample size, response frame, bias, measurement tools, response rate, statistical presentation, and participant description. Based on the scores obtained for these questionnaire characteristics, the percentage of methodological quality was obtained, with the current questionnaire being one of the 57 questionnaires rated as adequate out of a total of 164 reviewed questionnaires that were rated.

The questionnaire consists of 34 questions divided into three main sections. The first section collects personal, socio-demographic, and anthropometric data. The second section aims to assess the practice of the sport activity and its context. This section includes questions on topics such as competitive level, position, time and frequency of training, and type of diet. The third and final section relates to the consumption of DS and their possible effects on health or sports performance. It includes questions such as which DS they consume (See Supplementary Materials), why they consume them (“What are the reasons for your use of DS?” Health, sports performance, aesthetics…), who advises them to consume them (“Who motivated you to take DS?” Doctors, sports coach, dietician/nutritionist, friends, relatives…), where they usually buy them (“Where do you usually buy your supplements?” Pharmacies, specialist shops, internet…), when they take them (“Please indicate on which sports days you usually consume DS.” Training, competition, or both) and the specific timing (“Please indicate when you usually take DS” Before, during, or after training/competition). Accordingly, these questions allow an assessment of the prevalence and patterns of DS use among football players.

### 2.3. Statistical Analysis

The dependent variables of the study were the parameters related to the DS consumption of the football players and, as an independent variable, sex. Quantitative variables were presented as mean (standard deviation), and qualitative variables as percentages. The Shapiro–Wilk and Levene tests were used to test for normality and homoscedasticity, respectively. Differences between sex in quantitative variables were determined using a Student’s *t*-test or non-parametric tests if not following a normal distribution. Differences in qualitative variables were calculated using the chi-squared test of association (χ^2^). When significant differences were reported, the odds ratio (OR) was calculated.

The statistical level of significance was set at *p* < 0.05. The software programme JASP (version 0.17.3, Amsterdam, The Netherlands) was used for the statistical analysis of the data.

## 3. Results

Of the total sample, 96.12% reported having used DS at some time, with no significant differences between men and women (men: 95.65%; women: 97.30%; *p* = 1.000). In addition, 93.02% of the sample said they approved using DS for physical activity within the legal limits (males: 92.39%; females: 94.60%), with no differences between the sexes (*p* = 0.526) (Table 2).

Regarding the number of DS consumed, the total sample consumed an average of 5.62 (4.53) supplements, with 5.37 (4.71) for men and 6.24 (4.05) for women, with no differences between them (*p* = 0.110) (Table 2).

In general, the main reasons for consuming DS were to improve sports performance (90.32%) and for health reasons (21.77%), with no significant differences between the sexes (*p* > 0.05). The most common place to buy a DS was the Internet (33.07%), with differences according to sex (*p =* 0.004; OR = 0.23 [−2.49–−0.42]). Advice on the consumption of DS was mainly given by dieticians/nutritionists (75.00%). Significant differences between sex were found in the source of recommendation: dietician/nutritionist (*p* = 0.006; OR = 5.13 [0.37–2.90]) and physical trainer (*p* = 0.031; OR = 0.035 [−2.03–−0.072]) (Table 2).

In terms of the context in which DS was consumed, the most common situation in which it was consumed by participants was during training and competition (71.77%), with significant differences between sex (*p* = 0.023; OR = 3.21 [0.123–2.21]). With regard to the timing of consumption, the most common timing was before, during, and after exercise (48.39%.). There were significant differences between sex in those who consumed DS before exercise (*p* = 0.011; OR = 0.06 [−5.72–−0.04]) (Table 2).

**Figure 1:**
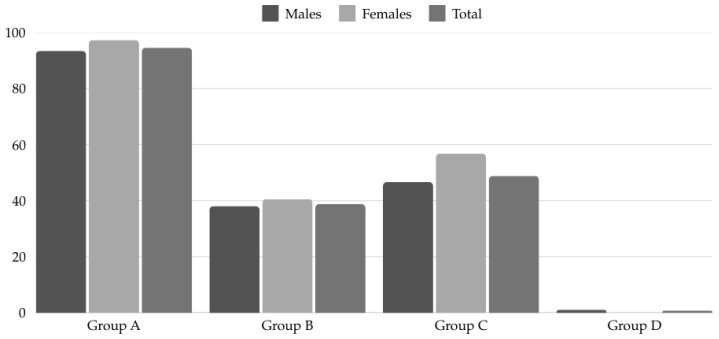
Dietary supplement consumption by sex according (%) to AIS classification.

**Figure 2:**
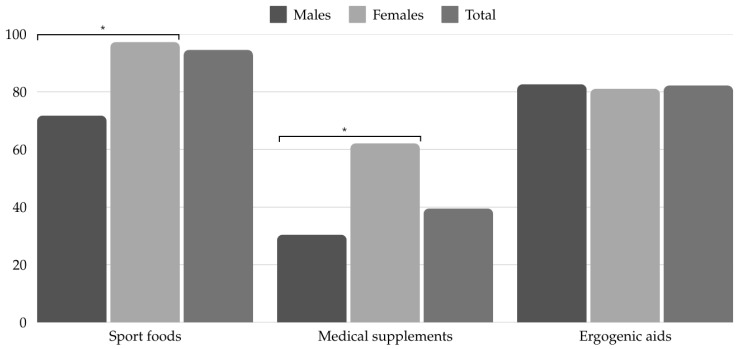
Consumption of dietary supplements in group A (AIS classification) by sex (%). Statistical difference between groups (*p* < 0.05) *.

According to the AIS classification of supplements, we found that the most consumed DS group overall was group A (94.57%), followed by group C (48.84%), group B (38.76%), and lastly group D (0.78%) (Figure 1). Within group A, ergogenic aids were most commonly used (82.71%). Statistically significant sex differences were found in the consumption of supplements in group A, specifically in sports foods (*p* = 0.034; OR = 3.25 [0.05–2.31]) and medical supplements (*p* < 0.001; OR = 3.75 [0.52–2.12]) (Figure 2).

The most frequently consumed DS in the sample were creatine (66.67%), caffeine (56.59%), whey protein (48.06%), isotonic drinks (37.21%), vitamin D (27.91%), energy bars (27.13%), magnesium (20.93%), and vitamin C (20.16%). Statistically significant sex differences were found for vitamin D (*p* = 0.04; OR = 3.27 [0.36–2.00]) (Table 3).

Participants rated their personal experience of the results they obtained from taking DS and rated the general use of DS. A scale of 1 to 5 was used, with 1 being the lowest score or ‘little result’ and 5 being the highest score or ‘a lot of result’. For the first variable, the mean score was 3.90 (0.87), with no significant differences found according to sex (males: 3.91 (0.91); females: 3.87 (0.80); *p* = 0.541). For the second variable, the mean of the responses was 4.12 (0.84), with no significant differences between sex (males: 4.18 (0.87); females: 3.97 (0.77); *p* = 0.110).

Regarding the use of diets, 63.57% reported that they followed some kind of diet. No significant differences were found between sex (*p* = 0.834), with 64.13% of men and 62.16% of women reporting following some kind of diet. The type of diet used by 48.78% of those who employed diet was ‘flexible’ (males: 49.15%; females: 47.83%), followed by 40.24% using a ‘Mediterranean’ diet (males: 40.68%; females: 39.13%), with no differences between the sexes (*p* > 0.05). Regarding the reason for following a diet, 69.51% of the football players stated that they did it for performance (males: 69.49%; females: 69.57%) and 28.05% for health (males: 28.81%; females: 26.09%), with no significant sex differences (*p* > 0.05). Participants reported being counselled mainly by dieticians/nutritionists (70.73%; males: 66.10%; females: 82.61%), followed by no counselling (18.29%; males: 22.03%; females: 8.70%), with no difference according to sex.

## 4. Discussion

This study aimed to evaluate and compare the consumption of DS in semi-professional male football players and professional female football players. Secondarily, we evaluated whether the participants were following any kind of diet. Although previous studies [2,4,14,23,24,25,26] have analysed the consumption of DS in male and female football players from different countries and levels of competition, to our knowledge, this is the first study that evaluates and compares the consumption of DS in male and female professional players from different Spanish football teams. The main findings of this study are (1) 96.12% of Spanish football players included in this study were found to consume DS; (2) sex differences were discovered in the pattern of DS consumption (type of DS consumed, place of purchase, source of recommendation, or time of consumption).

The high prevalence of DS use found in this study has also been reported in Turkish male and female players at different levels of competition (87.20%; males: 93.7%; females: 73.70%) [2] and in professional female footballers in Spain (84.10%) [12]. Other research has recorded a lower prevalence of DS, reporting consumption between 57.00% and 82.00% [14,23,24]. These differences could be related to the heterogeneity of the studies: the demographic make-up of the sample (age, sex, level of competition, etc.), the size of the sample, the year of the research, and the differences in football status between countries [2], as well as the evolution of football itself. In terms of sex differences, in our study, males and females reported similar levels of DS use. In contrast, Günalan et al. [2] reported higher use in male footballers, as did Baltazar-Martins [27] in a study involving 527 elite athletes from a variety of sports. Additionally, it has been stated that, generally, DS use is higher in men than in women [6]. We hypothesise that the increase in the professionalism and importance of women’s football in recent years, together with its augmented physical demands [28], may have contributed to the rise in DS consumption among women footballers, equating it to consumption in male footballers in our results.

Interestingly, in our study, we found that while 96.12% of the participants consumed DS, 63.57% followed some kind of diet. In this sense, it is essential for dieticians/nutritionists to verify that athletes are following a proper diet that covers the necessities before considering the use of DS, using a “food first, but not always food” approach [29]. Indeed, in the present study, the participants consumed an average of 5.62 supplements. Other research in football reported between 3 and 3.7 DS [23]. In other sports disciplines, results were variable: open water swimmers at different competitive levels had an average intake of 4.67 DS [30] and in amateur and professional rugby players, an average of 3.90 DS was reported [10]. This demonstrates a high consumption of DS in Spanish professional football, in both male and female categories, and therefore the need to monitor and assess DS use and use evidence-based approaches.

Athletes consume DS based on claimed or real benefits, such as correcting or prevent nutritional deficient, providing a convenient source of nutrients, or improving performance [6]. In the present study, the main reasons why footballers consumed DS were to improve sports performance (90.32%), to take care of their health (21.77%), and to improve their physical appearance (8.87%). These results are consistent with those reported by Günalan et al. [2], who found that the main reasons for use were to improve athletic performance (44.70%), to take care of health (20.60%), and to improve physical appearance (15.90%). Improving athletic performance was also found to be the main reason for use in other similar studies of football players (55.60–79.00%) [12,25,26]. This reason has been also reported in other sport disciplines. In contrast, Oliveira et al. [23] observed different motives for DS use among elite female footballers from different national teams: to stay healthy (66.00%), to speed up recovery (58.00%), and to increase energy or reduce fatigue (54.00%).

Football players can obtain numerous benefits from consuming DS, such as optimising performance in training and competition, preventing injuries, promoting recovery between training sessions, achieving optimal body weight and physical condition, and improving health status [1,4,7]. However, in order to use them correctly, it is important that a sports nutritionist carries out a risk–benefit analysis to determine which products are beneficial for each football player [6]. In the present study, dieticians/nutritionists were the main advisors of DS consumption (75.00%); therefore, a positive result was found. Along the same lines, in other similar studies, between 29.70% and 56.30% of football players were reported to be advised by a dietitian/nutritionist [2,31]. However, in other sports, the recommendation of DS consumption by these professionals did not exceed 20.00% of the participants [11,17,30]. Therefore, football players need to seek appropriate nutritional advice using evidence-based approaches [32]. Interestingly, our study also found that a higher proportion of female footballers (91.68%) were advised by dieticians/nutritionists compared to male footballers (68.18%). This finding is relevant, considering that it has been observed that athletes consume DS with higher scientific evidence when they are advised by a sport dietician/nutritionist as their main source of nutritional information [33].

It is essential to consider the influence of socio-economic and cultural factors on the decision to purchase DS. In particular, the ‘culture’ fostered within a particular sport plays an important role in shaping DS choices. This culture, influenced by coaches, managers and leaders within clubs and teams, varies widely between sports and countries. Such cultural dynamics may lead to focus on achieving the best performance, often without acknowledging the potential risks to athletes’ health or performance, including an increased likelihood of positive doping results [34]. Therefore, we believe it is crucial for future research to examine the impact of these factors on DS selection.

Regarding the place of purchase, it is crucial to choose a reliable source. However, in general, the regulation of DS is often lacking and varies from country to country [34], making it easier to buy DS from questionable sources [6]. In the present study, the most common places of purchase of DS were the Internet, specialised shops, and dieticians/nutritionists. We also found that men were more likely to buy online than women, and conversely, women consumed more DS provided by a club. Similarly, Molina et al. [12] reported that participants mainly purchased supplements online, in specialised shops, or through dieticians/nutritionists. Günalan et al. reported that the most common sources of DS were through clubs, pharmacies, and the internet. As in our study, men were found to purchase more from the internet than women [2]. Another study reported [23] that female football players most often purchased DS from shops, sponsors, and pharmacies. The present study, as well as the studies referenced herein, have not analysed the reliability of the place of purchase. This is a crucial aspect that should be addressed in future studies, as it is one of the most significant decisions to take into account when buying a DS [6].

In terms of DS consumption according to the AIS classification, the most consumed DS belong to group A, followed by group C, and group B. The results reported by Jiménez et al. [30] showed a high consumption of DS from groups A and C, ahead of group B. It is clear that it is necessary to offer nutritional education to football players to help them make better use of supplements, as in general, many athletes frequently consume DS without knowing their effects or risks [35]. Within group A, the most consumed subgroup was ergogenic aids, followed by sports foods, and finally medical supplements. In addition, women consumed more sports foods and medical supplements than men. This difference may be due to the fact that women tend to be more concerned about their health, aesthetics, and body composition [36].

UEFA’s expert group has published a nutritional guide, including dietary supplements that are potentially useful for football players. In terms of sports foods, they highlighted carbohydrate sources, such as drinks or gels, and protein sources, such as protein drinks. In terms of performance-enhancing supplements, they pointed out that creatine and caffeine are the supplements with a higher level of evidence, whereas beta-alanine or nitrates have limited evidence specific to football. Finally, they identified vitamin D, iron, and calcium as some of the most commonly required supplements [1]. In our study, we observed that the most commonly consumed DS were creatine monohydrate, caffeine, whey protein, sport drinks, vitamin D, energy bars, magnesium, and vitamin C. Günalan et al. [2] reported that the most frequently consumed DS were sports drinks, magnesium, vitamin C, vitamin D, and caffeine. Similar findings were reported by Aljaloud et al. [26] in male professional players from three Saudi Arabian teams, with sports drinks being the most commonly consumed DS, followed by vitamin C, caffeine, iron and omega-3. In elite female footballers from different national teams, the main DS consumed were vitamin D, omega-3, protein, vitamin C, and energy drinks [23], whereas in Spanish footballers, whey protein, sports drinks, creatine monohydrate, energy bars, and caffeine predominated [12]. On the other hand, among Spanish athletes from different sports, the most common supplements observed were protein, amino acids, multivitamins, glutamine, and creatine monohydrate [27].

In our research, the two most frequently used supplements were creatine monohydrate and caffeine, both of which are group A ergogenic aids. These are supplements with strong evidence, so their widespread use in football players [2,12,26] may be due to the safety of their use and the associated effects on athletic performance [6,8]. Other group A ergogenic aids with a low prevalence of use in football players were beta-alanine or nitrates [2,12,23,26]. This may be because caffeine and creatine have more evidence of efficacy in football than beta-alanine or nitrates [1].

Among sports foods, our data and those of other studies show that sports drinks and proteins are the most frequently consumed DS by football players [2,12,23,26]. Given the significant multiple benefits of both products, providing a convenient form of energy and nutrients [6] in a population with increased requirements [1], their high prevalence in this population is logical.

With regard to vitamin D, it was the most commonly consumed supplement in our and other studies [2,23], and was reported to be most frequently consumed by women. These findings are consistent with similar studies reporting that women generally use more medical supplements than men, highlighting that woman are more concerned with health than performance [37]. This difference may also be related to the prevalence of vitamin D deficiency, as vitamin D deficiency has been shown to be more common in women than in men [38]. An important factor observed in our results that may influence this difference is the fact that women are more likely to seek advice from health professionals (i.e., nutritionists) than men. In fact, among a sample of eight elite women’s national teams, vitamin D was the most commonly used supplement, with health professionals providing more advice than coaches/physical trainers [23].

In fact, vitamin D is one of the nutrients that most needs to be supplemented in athletes and in the general population [6]. In particular, a high prevalence of vitamin D deficiency and insufficiency has been reported in professional football [39,40]. Therefore, it is important to test footballers regularly to identify any deficiencies, which should be treated by professionals [1].

Another of the most commonly consumed DS was magnesium, in line with Günalan et al. [2], where it was the second-most consumed supplement. This is a group C supplement according to the AIS classification, among the supplements with no proven evidence. Consequently, its high consumption could be related to a popular use based on the fact that this mineral is essential and is involved in muscle actions, energy metabolism, and cardiorespiratory function, as hypothesised by Günalan et al. [2].

Finally, we reported that vitamin C was consumed by more than 20.00% of the participants in our study, and it was also one of the five most frequently used supplements in three other studies of football players [2,23,26]. It has also been reported as one of the most used supplements in other sports, such as swimming [30,41]. Despite the possible beneficial effects of vitamin C supplements, it should be noted that high doses may improve training adaptations and thus be more harmful than beneficial [42,43].

The consumption of DS is not free from risks, particularly when consuming supplements which lack evidence of efficacy (i.e., group C from AIS classification). Despite their potential benefits, the use of DS may be associated with negative effects on performance and health, in addition to the possibility of a positive result in a doping test. To mitigate these risks, it is relevant to analyse the evidence of the effectiveness and safety of a DS. Additionally, it should be ensured that DS are provided from a reliable source to avoid the risk of contamination with banned substances [6]. Our results showing that 48.84% of the football players consumed supplements from Group C emphasize the lack of knowledge and the need for professional advice.

### Strengths and Limitations

One of the key strengths of this research is the use of a questionnaire specifically designed and validated by a team of experienced sport scientists [22]. Its methodological quality has been rigorously evaluated through a systematic review and meta-analysis [9], enhancing the reliability of the results. Furthermore, the questionnaire has been consistently applied across various sports settings [10,11,16,17,18,19,20,21], further supporting its validity and applicability. Finally, we emphasise the relevancy of our results, as Spanish football is internationally recognised as one of the most important and successful in the world [1,44,45].

This study has different limitations. Firstly, the number of female participants is limited, which is related to the difficulty in accessing the sample; this issue limits the generalisability of the conclusions. To enhance the comparison between sex in future studies, a proportional sample of male and female participants should be recruited. However, the distribution of participants in this study is consistent with the overall demographic composition of males and females in Spain, taking into account the inherent differences in the number of players across different leagues. Also, as a study based on self-report analysis, some questions are subject to the subjects’ perceptions and interpretations. Self-reported data, particularly in relation to diet, can be subject to various types of bias, such as response bias, recall bias, or social desirability bias [46], which can reduce the reliability of the data obtained. In addition, anthropometric data were self-reported and relevant variables such as body fat were not obtained. Likewise, it would have been interesting to include a more detailed analysis of the consumption of DS, including aspects such as quantity, time of consumption, and form of consumption, among others. This aspect is important to consider, as when a DS is not consumed following the correct protocol, it can have a negative impact on athletes’ health and performance, even when the DS has an adequate level of scientific evidence. Furthermore, future studies should incorporate an analysis of socio-economic and cultural factors, as well as examine the role of sports club policies and sponsorships. Indeed, the role of sport sponsorship and sports policy has been speculated to have an impact on DS use, but has not been explored in depth in our or previous studies [2,33]. Together, these aspects may significantly influence dietary supplement consumption patterns.

## 5. Conclusions

The present study shows a high prevalence of DS consumption in Spanish male and female football players, higher than reported in other studies. According to AIS classification, the participants mainly consumed group A supplements, followed by group C. Specifically, the most used supplements were creatine, caffeine, whey protein, isotonic drinks, vitamin D, energy bars, magnesium, and vitamin C. The main reason associated with supplement consumption was performance enhancement.

Considering that some of the most commonly used DS have a low level of evidence tied to them, it is important that football players receive nutritional education on DS use, which should be provided by the appropriate professionals: sports nutritionists.

In terms of sex differences in supplement consumption, differences were observed in the consumption of specific groups of supplements, such as medical supplements (higher consumption in women) or sports foods (higher consumption in men), as well as in the place of purchase or consumption situation.

## Figures and Tables

**Table 1:** Participants characteristics.

Variable	Males (*n* = 92)	Females (*n* = 37)	Total (*n* = 129)	*p*-Value
Age (years)	26.73 (4.28)	26.57 (5.12)	26.68 (4.52)	0.786 ^a^
Body weight (kg)	74.62 (5.72)	58.26 (5.35)	69.93 (9.30)	<0.001 * ^b^
Weekly training sessions (days)	5.62 (0.72)	5.78 (0.67)	5.67 (0.71)	0.285 ^a^

All values show the mean (standard deviation); Inter-group differences were calculated by Mann–Whitney U test (^a^) and an independent *t*-test (^b^). Statistical difference between groups (*p* < 0.05) *.

**Table 2:** Sex differences in patterns of dietary supplement use.

		Male (*n* = 88)	Female (*n* = 36)	Total (*n* = 124)	*p*-Value ^a^
Main consumption motive	Performance	90.91%	88.89%	90.32%	0.991
	Healthcare	25.00%	13.90%	21.77%	0.174
	Improvement of physical appearance	6.82%	14.29%	8.87%	0.363
	Nutritional deficit	6.81%	5.57%	6.45%	1.000
Most common site of purchase	Internet	40.91%	13.89%	33.07%	0.004 *
	Specialized shop	28.41%	33.33%	29.84%	0.586
	Dietician/Nutritionist	27.27%	36.11%	29.84%	0.329
	Club	2.27%	25.00%	8.87%	<0.001 *
Source of recommendation	Dietician/Nutritionist	68.18%	91.68%	75.00%	0.006 *
	Physical trainer	36.36%	16.67%	30.65%	0.031 *
	Team mate	20.46%	11.11%	17.74%	0.216
	Coach	10.28%	8.33%	9.68%	1.000
Situation of consumption	Training + competition	65.91%	86.11%	71.77%	0.023 *
	Training + competition + rest	14.77%	2.78%	11.29%	0.109
	Training	11.36%	5.56%	9.67%	0.510
	Competition	7.96%	5.56%	7.26%	0.931
Timing of consumption	Before + during + post	44.32%	58.33%	48.39%	0.156
	Post	27.28%	36.11%	29.84%	0.329
	Before	19.32%	0.00%	13.71%	0.011 *
	Indistinct	6.82%	5.56%	6.45%	0.795

Inter-group differences were calculated by chi-square test ^a^. Statistical difference between groups (*p* < 0.05) *.

**Table 3:** Most commonly consumed dietary supplements according to sex based on AIS classification.

Group AIS		DS	Males (*n* = 92)	Females (*n* = 37)	Total (*n* = 129)	*p*-Value ^a^
Group A	Sports foods	Whey protein	45.65%	54.05%	48.06%	0.388
		Sport drinks	34.78%	43.24%	37.21%	0.369
		Sport bars	27.17%	27.03%	27.13%	0.986
		Energy gels	15.22%	21.62%	17.05%	0.382
		Carbohydrates “gainers”	13.04%	10.81%	12.40%	0.958
		Vegan protein	3.26%	10.81%	5.43%	0.200
	Medical supplements	Vitamin D	20.65%	45.95%	27.91%	0.004 *
		Multivitaminic	11.96%	16.22%	13.18%	0.719
		Iron	6.52%	16.21%	9.30%	0.168
		Probiotics	3.26%	10.81%	5.43%	0.200
	Ergogenic aids	Creatine monohydrate	69.57%	59.46%	66.67%	0.271
		Caffeine	56.52%	56.76%	56.59%	0.981
		B-alanine	8.70%	16.22%	10.85%	0.214
Group B		Vitamin C	21.74%	16.22%	20.16%	0.479
		Omega 3	14.13%	10.81%	13.18%	0.829
		Curcumine	7.61%	18.92%	10.85%	0.353
Group C		Magnesium	21.74%	18.92%	20.93%	0.722
		Glutamine	15.22%	10.81%	13.95%	0.710
		Melatonine	9.78%	16.22%	11.63%	0.467
		BCAA	9.78%	16.22%	11.63%	0.467
		Vitamin E	8.70%	8.11%	8.53%	1.000
		EEAA	5.44%	10.81%	6.98%	0.483

BCAA (branched chain amino acids), DS (dietary supplements), EEAA (essential amino acids). Inter-group differences were calculated by chi-square test ^a^. Statistical difference between groups (*p* < 0.05) *.

## Data Availability

The data that support the findings of this study are available from the corresponding author upon reasonable request.

## Supplementary Materials

The following supporting information can be downloaded at: https://www.mdpi.com/article/10.3390/nu17040602/s1, Table S1: List of evaluated dietary supplements.
