# Supplementary material for: Dietary Supplement Use in Competitive Spanish Football Players and Differences According to Sex
Source: Nutrients. 2025 Feb 7;17(4):602. doi: 10.3390/nu17040602 (PMC11858016; doi:10.3390/nu17040602)
Supplement: Supplementary file 1 [file nutrients-17-00602-s001.zip › nutrients-3428139-supplementary.pdf]

**Supplementary material**

**Table S1. List of evaluated dietary supplements:**

| | | | | |
|---|---|---|---|---|
| 5-HTP (5-Hydroxytryptophan) | Beta-Alanine | Creatine (Ethyl Ester) | Isomaltulose | Synephrine (p-synephrine) |
| ATP | Bicarbonates | Curcumin | Royal Jelly | Tart Cherry Supplement |
| Aspartic Acid | Carbohydrate Blockers | Dextrose | Soy Lecithin | Taurine |
| Alpha Lipoic Acid (ALA) | Caffeine | Dimethylglycine | Leucine | Theanine |
| Phosphatidic Acid | Carbohydrates ("Gainers") | Diuretics | Brewer's Yeast | Theacrine |
| Hyaluronic Acid | Carnitine (Acetyl-L-Carnitine) | Electrolytes (in powder or pills) | Magnesium | Green Tea (whole or extracts) |
| Omega-3 Fatty Acids | Carnitine (L-Carnitine) | Epicatechin | Maltodextrin | Tribulus |
| Omega-6 Fatty Acids | Shark Cartilage | Epigallocatechin -3-gallate (EGCG) | Melatonin | Tyrosine |
| Omega-9 Fatty Acids | Micellar Casein | Spirulina | Methylsulfonyl methane (MSM) | Medium-Chain Triglycerides (MCT) |
| Conjugated Linoleic Acid (CLA) | Chitosan | Pre-Workout Formulas | Nitrate (beetroot juice) | Green Tea (whole or extracts) |
| Coconut Oil | Cyclodextrins | Sodium Phosphate | Nootropics | Vitamin C |
| Cod Liver Oil | Zinc | Ginseng | Chromium Picolinate | Vitamin D |
| Flaxseed Oil | Arginine | Glycerol | Testosterone-Boosting Pollen | Vitamin E |
| Evening Primrose Oil | Citrulline (Malate or L-Citrulline) | Glucosamine | Hormonal Precursor | Vitamin K |
| Primrose Oil | Mineral Complex | Glutamine | Probiotics | Yohimbine |
| Amylopectin | Arginine | Greens | Beef Protein | ZMA |
| Essential Amino Acids (EAA) | Vitamin Complex | Guarana | Whey Protein | Vitamin C |

| | | | | |
|---|---|---|---|---|
| Branched-Chain Amino Acids (BCAA) | Chondroitin | Casein Hydrolysate | Vegetable Protein (soy, hemp, etc.) | |
| Energy Bars | Creatine (Monohydrate) | Hydroxymethyl butyrate (HMB) | Quercetin | |
| Isotonic Drink | Creatine (Kre-Alkalyn) | Iron | Ribose | |
